# Oscillatory Patterns of Phase Cone Formations near to Epileptic Spikes Derived from 256-Channel Scalp EEG Data

**DOI:** 10.1155/2018/9034543

**Published:** 2018-11-21

**Authors:** Ceon Ramon, Mark D. Holmes, Mackenzie V. Wise, Don Tucker, Kevin Jenson, Samuel R. Kinn

**Affiliations:** ^1^Department of Electrical and Computer Engineering, University of Washington, Seattle, WA 98195, USA; ^2^Regional Epilepsy Center, Department of Neurology, University of Washington, Seattle, WA 98195, USA; ^3^Department of Psychology, University of Oregon, Eugene, OR 97403, USA; ^4^Brain Electrophysiology Lab Co., Eugene, OR, USA

## Abstract

Our objective was to determine if there are any distinguishable phase cone clustering patterns present near to epileptic spikes. These phase cones arise from episodic phase shifts due to the coordinated activity of cortical neurons at or near to state transitions and can be extracted from the high-density scalp EEG recordings. The phase cone clustering activities in the low gamma band (30–50 Hz) and in the ripple band (80–150 Hz) were extracted from the analytic phase after taking Hilbert transform of the 256-channel high density (dEEG) data of adult patients. We used three subjects in this study. Spatiotemporal contour plots of the unwrapped analytic phase with 1.0 ms intervals were constructed using a montage layout of 256 electrode positions. Stable phase cone patterns were selected based on the criteria that the sign of the spatial gradient did not change for at least three consecutive time samples and the frame velocity was within the range of propagation velocities of cortical axons. These plots exhibited dynamical formation of phase cones which were higher in the seizure area as compared with the nearby surrounding brain areas. Spatiotemporal oscillatory patterns were also visible during ±5 sec period from the location of the spike. These results suggest that the phase cone activity might be useful for noninvasive localization of epileptic sites and also for examining the cortical neurodynamics near to epileptic spikes.

## 1. Introduction

During interictal periods, epileptiform spikes can be observed in scalp EEG data. When present, these discharges are highly correlated with the occurrence of clinical seizures. The mechanism of the formation of these epileptic spikes could be related to the interconnected activity of cortical neurons. In other words, it is a possibility that a group of neurons are taking part in a coordinated fashion to form an epileptic spike. Analysis of phase cone [1–3] formations near to epileptic spikes derived from the high-density, 256-channel, scalp EEG has the potential to explore and identify this coordinated cortical activity. By using this technique, our results indicate that there is a connection between phase cone formations and epileptic spike activity and this finding will be very useful for studying the spatiotemporal behavior of the formation and dissipation of epileptic spikes in scalp EEG data.

The concepts of EEG phase cones were introduced by Freeman and his colleagues while analyzing the spatiotemporal plots of the phase derived from neocortical EEG recordings of the rabbit brain [1–3]. The rabbit data were collected with an array of 64 electrodes (8 × 8 grid, 7 × 7 mm size, with 0.79-mm interelectrode distance). These spatial phase plots had maxima and minima which could be best described by the shape of cones and these cones rapidly appeared and disappeared. The size of the phase cones also varied from two electrodes to all the way to 8 × 8 grid electrodes. A newer procedure based on conditional probabilities has also been developed to optimize the detection of phase cones in the visual cortex of the above described rabbit EEG data [4].

In spatiotemporal frames one can see the amplitude and phase modulated waves in theta (3–7 Hz) and alpha (3–12 Hz) bands with carrier frequencies in beta (12–30 Hz) and gamma (30–80 Hz) bands [4–6]. Later on, similar phase cone patterns were also observed from intracranial grid recordings of a patient [7]. This data set was collected with an 8 × 8 microgrid array made of 0.5 mm stainless steel wires spaced 1.25 mm apart (aperture 10 × 10 mm). Aperiodic phase resetting at alpha and theta rates were also observed in human scalp EEG data collected with a curvilinear array of 64 electrodes, 3 mm apart, extending 18.9 cm across the scalp [8]. Similar techniques have also been applied to look for phase cones from high-density, 256-channel, scalp EEG data of epilepsy patients, and it was found that the rate of formation of phase cones in the low gamma (3–50 Hz) band was higher in epileptogenic zones [9]. The minimum size of phase cones derived from 256-channel scalp EEG data was between 2 and 4 cm [9] based on the interelectrode separation of about 2 cm [10]. All of these findings show that EEG phase cones can be observed from intracranial as well as from scalp recordings. An excellent description of phase cones is also available on the Internet (http://www.scholarpedia.org/article/Freeman's_mass_action).

These phase cones arise due to the coordinated activity of cortical neurons in a local neighborhood at mesoscopic (∼0.5–1.0 mm) scales in the brain. On the neocortical surface, microgrid electrodes of 0.5 mm diameter wires are sufficient to pick up this coordinated activity of cortical neurons located within 4–6 hypercolumns (∼10^5^ to 10^6^ neurons) of the neocortex [5, 7]. This coordinated activity gives rise to episodic phase jumps which can be observed on a single or multiple nearby electrodes of a microgrid on the cortical surface. These episodic phase jumps at mesoscopic scales will also give rise to neuronal pulse trains in local areas of the neocortex (∼1 cm or larger) through cortical network connections, and looking on the cortical surface, one could see spatiotemporal patterns of standing or traveling waves along with waxing and waning of phase cone structures in theta and alpha bands [3, 5, 7, 11]. If the coordinated activity of neurons spreads in larger areas (∼2 cm or more) of the neocortex, one would be able to observe it on two or more electrodes of high-density (256-channel) scalp recordings where interelectrode separation is about 2 cm [10]. This is probably what we are seeing in the results reported here and in our previous works [9, 12].

These phase cones are related to state transitions in the dynamics of cortical networks at mesoscopic (∼0.5–1.0 mm) scales in the brain [3, 6] and possibly could also be related to neural avalanches at microscopic scales [13]. The dynamical nature of phase cones is very similar to the formation and dissipation of bubbles in a pot of boiling water or cascades of sand piles during metastable states [7, 14]. These cortical networks operate at or near to a critical metastable state, and an external or internal stimulus could trigger a phase transition. This could happen at microscopic, mesoscopic, or at global scales in the neocortex. This represents a scale-free property of the cortical neurodynamics. This multilevel dynamics has been used to study the animal behavior [15], formation of epileptic seizures [16], and the sustained levels of wakefulness in humans [17]. This is a brief review of the concepts of EEG phase cones and their possible applications to brain behavior and epilepsy localization.

## 2. Materials and Methods

### 2.1. Patient Data

Data sets of three subjects were used for this study, who were all patients at the Harborview Medical Center, University of Washington for presurgical epilepsy monitoring. All data sets were collected with the approved Human Subjects Guidelines at the University of Washington. Details of these data sets are described below.

**Figure 1:**
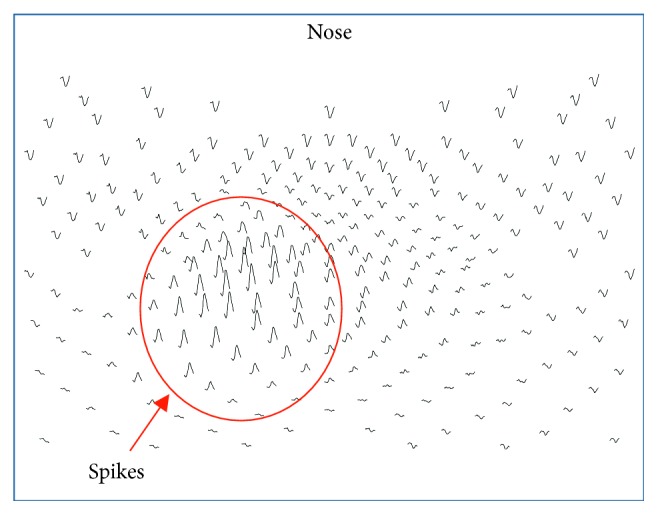
Topographic plot of the 256-channel scalp EEG data showing the location of the spike in the left central and parietal areas.

The subject #1 was an adult thirty-five years old female who was a candidate for surgery. Prior to invasive recordings, during presurgical evaluations, high-density 256-channel scalp EEG data were collected continuously for one-and-a-half hour during awake and sleep states. The data were collected with an EEG system developed by Electrical Geodesics, Inc. (EGI), Eugene, OR, USA. Now, it is acquired by Philips and known as EGI-Philips (https://www.egi.com/). The electrode caps were filled with a conducting gel with an effective diameter of approximately 1.0 cm. Thus, the effective electrode diameter becomes 1.0 cm. For an adult head, from the center of one electrode to the other, the interelectrode separation is approximately 2.0 cm [10]. The data were collected with a sampling rate of 1.0 KHz, i.e., the time difference between two consecutive samples was 1.0 ms. In the waking state, there was a symmetric 9-10 Hz posterior alpha rhythm that attenuated symmetrically with eye opening. A central mu rhythm (8–13 Hz) was seen on either side from time to time. Drowsiness was characterized by waxing and waning of the alpha rhythm, slow lateral eye movements, and intermittent generalized theta activity. Stage II sleep was characterized by symmetric V-waves, K-complexes, sleep spindles, and positive occipital sharp transients of sleep. Interictal epileptiform abnormalities (spike and sharp waves) were observed, mainly during drowsiness and stage II sleep. These discharges were localized over left central-parietal areas. A spatiotemporal plot of an epileptic spike is given in Figure 1, which shows the spike activity in central and parietal areas of the brain. It is based on the montage layout of 256 electrode positions. The nose is on the top in all of these spatial plots in this report.

Interictal invasive subdural ECoG (electrocorticograms) were also collected later on with an 8 × 8 grid electrodes and strip electrodes on left temporal, central, and parietal areas. The electrodes on the grid had an exposed surface area defined by 2.3 mm diameter and with center-to-center, interelectrode separation of 1.0 cm [18]. The epileptic spikes and seizures were observed over left central-parietal areas, similar to what was observed in the high-density scalp recordings given in Figure 1. Anatomical MRI data were also collected.

The subject #2 was an outpatient adult male whose dEEG data during sleep were collected with similar protocol as described above for the subject #1. This data set was collected for monitoring the interictal and epileptic spike activity. The dEEG was recorded over two separate days, two months apart. The total duration of the data was four hours. The dEEG recordings received visual clinical analysis for manual interictal spike detection and also with an automated spike detection software, Spike Beacon, from EGI. These two modes of detection combined with detailed temporal-spatial analyses reveal the right basal temporal lobe and the right lateral temporal lobe to be the foci of this subject's interictal spikes. Both of these right temporal neocortical regions combine temporally to create a complex network with a spread of activation that begins in the basal temporal lobe (seen at the peak of the prodromal discharge, −152 ms, and seen at the onset of the interictal spike, −37 ms) that then spreads to the right lateral temporal lobe (seen at the spike peak, 0 : 00 ms).

The subject #3 is an adult twenty-one-year-old female whose dEEG data were collected as an outpatient for epilepsy monitoring. This data set was also four hours long collected in two separate sessions, two months apart. Both recordings received visual clinical analysis for manual interictal spike detection and automated spike detection with the Spike Beacon software (EGI) to establish a standardized spike count. The two modes of detection combined with detailed temporal-spatial analyses revealed the left lateral-ventral prefrontal cortex and left premotor area to be the foci of this patient's dominant interictal discharges. Both areas combine temporally to create a complex network of temporal propagation from the left lateral-ventral prefrontal cortex (seen at spike onset) to the left premotor area over a 10 ms period leading to the spike peak (0 : 00 ms). Spatiotemporal plots of the scalp potentials revealed the spreading of the activity from left premotor area to the left central and parietal midline area.

### 2.2. Data Analysis

Artifact-free high-density raw EEG data during the sleep period were searched and selected for analysis. For the subject #1, we found a ten-minute-long continuous EEG data which had epileptic spikes but did not have large motion related and muscle artifacts. This data had eleven well-recognizable epileptic spikes at irregular intervals, out of which three spikes were selected for further analysis when the subject was sleeping. The selected spikes were roughly two minutes apart from each other without any epileptogenic activity in between the spikes. The two-minute gap between spikes is ample time for cortical neurons to return to the stable state if they were involved in the previous epileptic spike activity. A ±5 sec long data segment from the location of the selected spike was used to study the behavior of cortical neurons near to that particular spike.

Most of the analysis was carried out using the EEGLAB software. The ten-minute long raw data were filtered in 0.3 to 300 Hz passband to reduce the visible muscle artifacts. After that, the CleanLine (http://www.nitrc.org/projects/cleanline/) software as a plugin in EEGLAB was used to remove the 60 Hz power line artifacts from the band-passed filtered data. In addition, harmonics of 60 Hz at 120 Hz, 180 Hz, and 240 Hz were also removed. After that, the principal component analysis (PCA) was applied to the data using EEGLAB software to remove muscle artifacts. We computed 64 principal components for the 10 min-long data out of which eight components were identified as eyeblink and muscle activity-related components and were removed. The PCA-pruned data were saved for further analysis.

The data analysis was the same for each epileptic spike. Our procedures for one of the spikes are described here. The EEG phase cone analysis was performed in the low gamma (30–50 Hz) band and in the ripple band (80–150 Hz). The PCA-pruned data were filtered in the appropriate EEG band with an equiripple FIR (finite impulse response) Parks-McClellan filter. The ripples in the passband were limited to 0.05 dB in order to reduce ripples-related artifacts in the filtered data. The Hilbert transform was applied to compute the analytic phase. The phase of the analytic signal has a sawtooth pattern which was unwrapped to produce a cumulative linear phase of the signal, and then it was detrended by use of the first difference method. The instantaneous phase frequency, *F*_*i*_, was computed from this detrended phase. These procedures have been used earlier by us [9, 12]. The *F*_*i*_ was limited to 30–50 Hz while searching for the stable phase cone patterns in the low gamma band. Similarly, the *F*_*i*_ was limited to 80–150 Hz while searching for the stable phase cone patterns in the ripple band. The rate of change in the phase with distance (rad/mm) was computed from the spatial location of electrodes on the scalp.

Several criteria were applied to select stable phase cone patterns. These included the following: (1) *F*_*i*_ was within the temporal band, e.g., 30–50 Hz for low gamma band, (2) sign of the spatial gradient and maxima or minima did not change for at least three time samples, and (3) the frame velocity should be within the range of conduction velocities of cortical axons, 1–10 meters/sec [19–23]. However, one should note that measured maximum value of the propagation of phase gradients in rabbit data is about 2 meters/sec and can cover cortical areas of size of several cm^2^ to form stable phase cones [2, 8, 24]. A summary of sequential steps, i.e., pipeline, to look for stable phase cones frames is as follows:

1. Filter the EEG data in the appropriate band, e.g., low gamma band
2. Compute phase using Hilbert transform, unwrap the phase and detrend
3. Construct spatial phase cone patterns based on the spatial location of electrodes
4. Selected frames containing phase cones which are within the selected frequency band and also the signs (+or −ve) of spatial gradient did not change for at least three consecutive time steps
5. Apply physiological constraint, i.e., frame velocity < conduction velocity (1–10 m/s)

**Figure 2:**
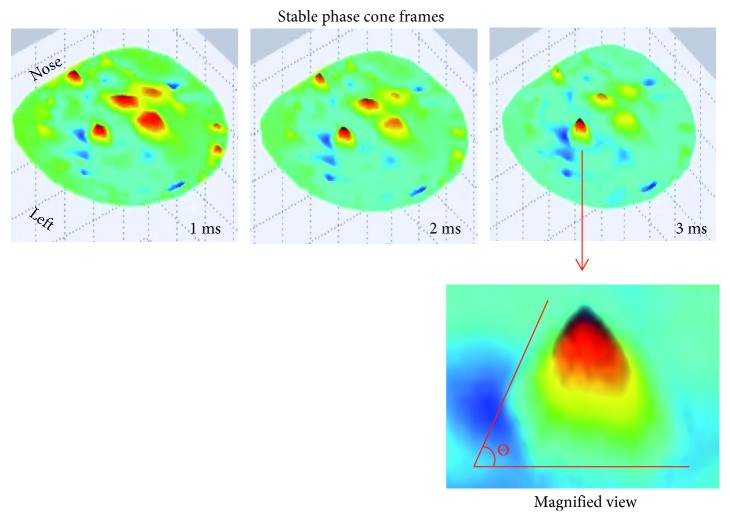
Stable phase cone patterns in three consecutive time frames. Lower plot shows the phase cone angle which also should not change in the above three frames.

A pictorial representation of the formation of stable phase cones is given in Figure 2. The phase cones are shown in three consecutive time frames of 1.0 to 3.0 ms. These are constructed from the unwrapped phase extracted from the EEG data and then plotted with a montage layout of 256 electrode positions. More details are given below. A detailed view of the spatial shape of the phase cone with shape angle, *θ*, is also shown. For a stable phase cone, the shape angle, *θ*, should not change in three consecutive frames.

Based on these above-described criteria, a stable cluster of phase cones near to the spike was computed. Here, the term cluster is used in the spatial sense on the scalp surface. The analysis was performed over a period of ±5 sec from the location of the spike. The rate of formation of phase cones per second was computed with a resolution of one second over a period of ±5 sec, or more precisely, from −5 sec to +6 sec period. Color intensity plots of the rate of formation of phase cone clusters were constructed using a montage of the layout of 256 electrode positions and displayed as if one was looking on top of the subject's head.

Similar procedures for data analysis were used for subjects #2 and #3. Three spikes were randomly selected in two hours long data which were free from artifacts and then the phase cones were computed over a ±5 sec period from the location of each spike.

### 2.3. Shuffled Data

**Figure 3:**
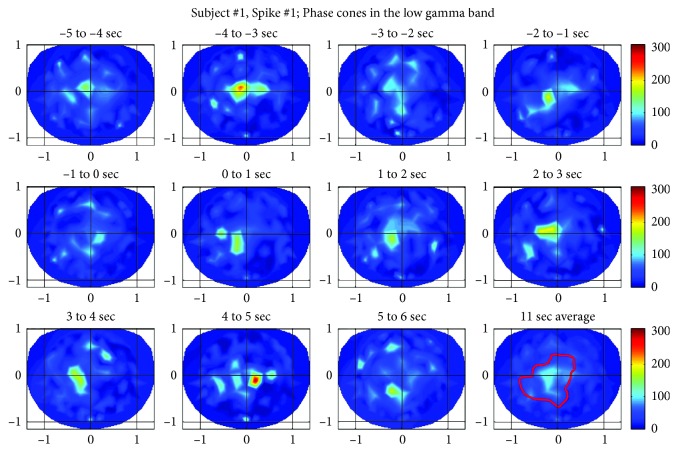
Phase cone patterns in the low gamma band near to the epileptic spike #1 of the subject #1. Last plot (lower right) shows the average over the eleven second period. Red contour line defines the possible area of the epileptic spike activity.

**Figure 4:**
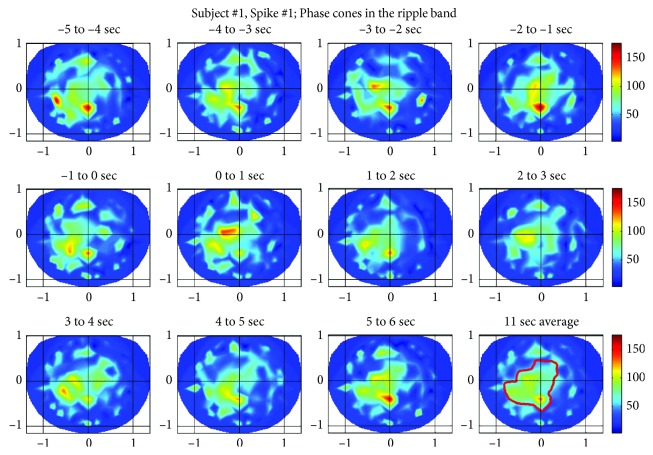
Phase cone activity in the ripple band for the spike #1 of subject #1.

**Figure 5:**
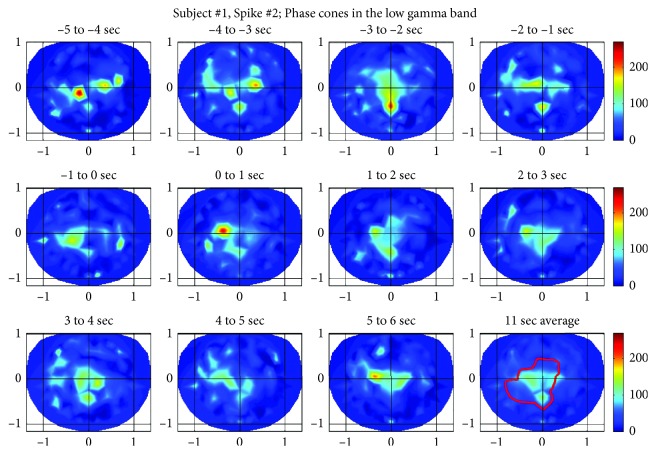
Phase cone activity for the spike #2 of the subject #1 in the low gamma band.

**Figure 6:**
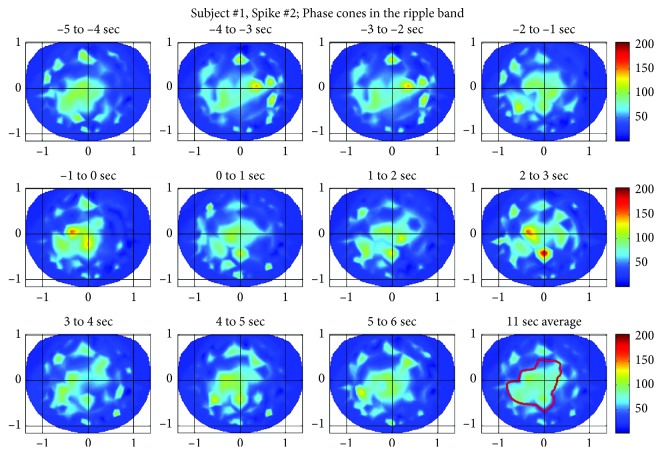
Phase cone activity in the ripple band for spike #2 of the subject #1.

**Figure 7:**
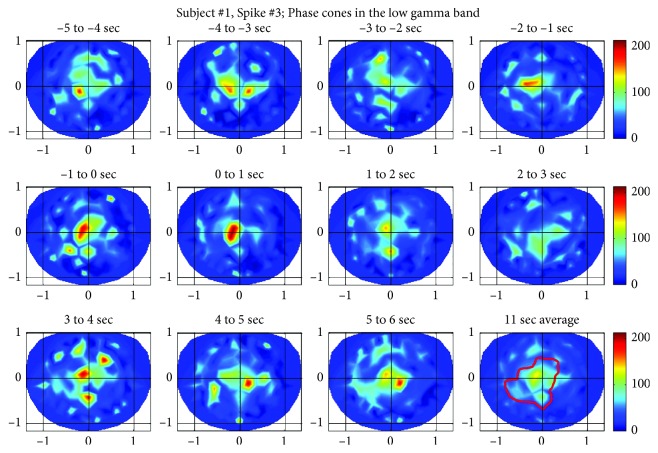
Phase cone activity in the low gamma band for the spike #3 of the subject #1.

**Figure 8:**
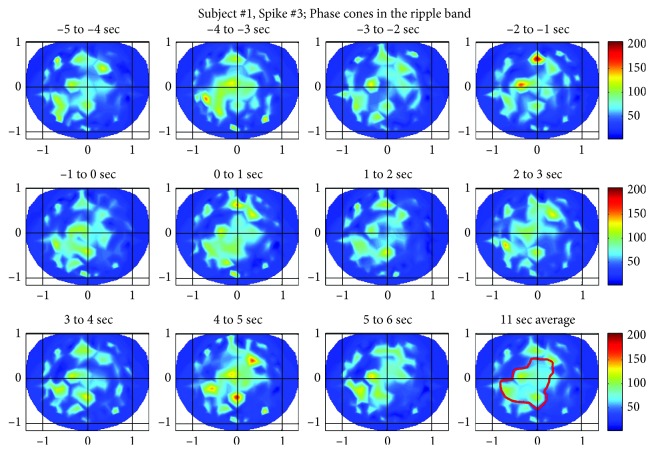
Phase cone activity in the ripple band for the spike #3 of subject #1.

**Figure 9:**
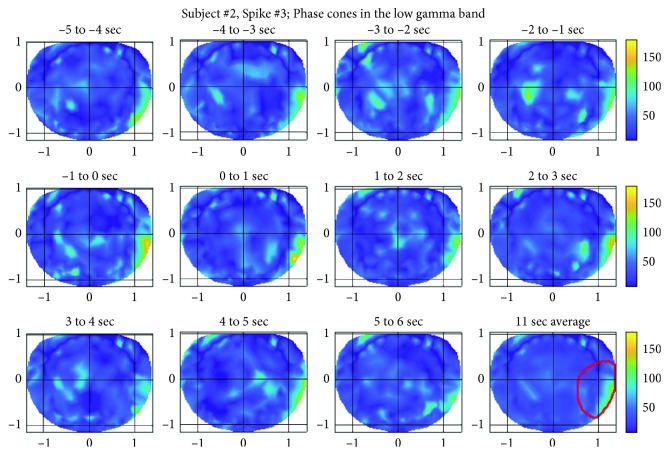
Phase cone activity in the low gamma band for the subject #2.

**Figure 10:**
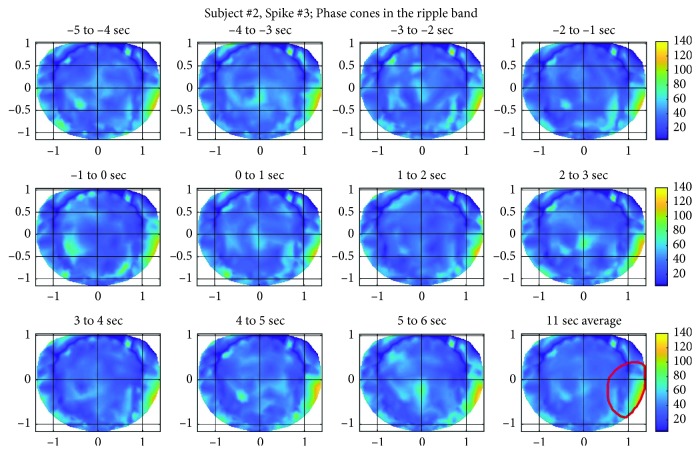
Phase cone activity in the ripple band for the subject #2.

**Figure 11:**
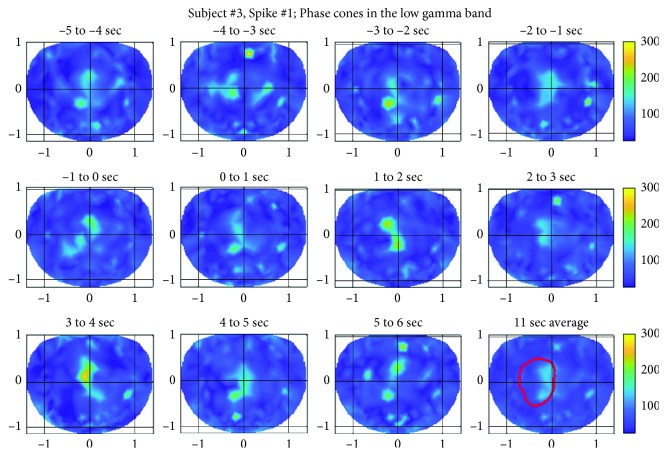
Phase cone activity in the low gamma band for the subject #3.

**Figure 12:**
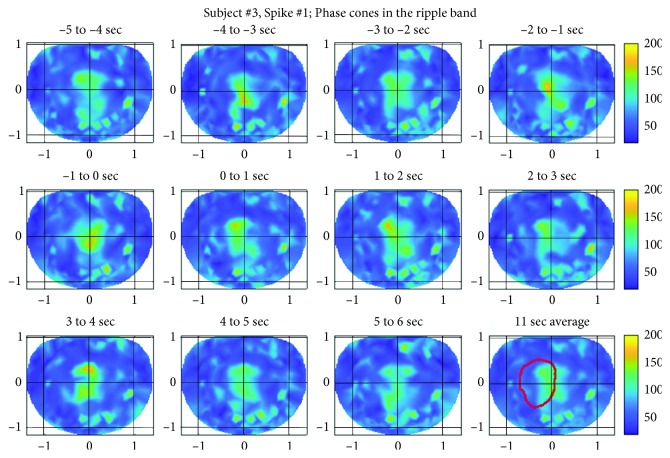
Phase cone activity in the ripple band for the subject #3.

A ten-minute long, 256-channel EEG data was converted to a row vector, randomly shuffled using “ramdperm” command in Matlab and then rearranged as a 10-minute long 256-channel EEG data. Out of this, one-second long data segments were randomly selected and then used to compute the rate of formation of phase cones in the low gamma band and in the ripple band. This process was repeated for 100 trials. The averaged values of the rate of phase cone formations over 100 trials were 13 ± 2.3 (*n* = 100) for the low gamma band and 17 ± 4.6 (*n* = 100) for the ripple band. These values for randomly shuffled data are for reference only and have not been subtracted in all the plots given in the results section. These values are much lower as compared with the actual rate (100–250 phase cones/sec) of phase cone formations near to epileptic spikes area as shown in Figures 3–12. Thus, one can consider that the rate of phase cone formations near to epileptic spikes is significantly higher as compared with the randomly shuffled noise-like data.

## 3. Results

### 3.1. Subject #1

#### 3.1.1. Spike #1 of the Subject #1

The rate of formation of phase cones in the low gamma band for the spike #1 is given in Figure 3. In these spatial plots, the nose is on the top, back of the neck is at the bottom, left of the subject is on the left side of the plot, and right side of the subject is on the right side of the plot. The horizontal and vertical axes are in normalized length units. Plots are with one-second resolution starting with −5 to −4 sec, −4 to −3 sec, and so on. The location of the beginning of the spike is at 0.0 sec, and in general, the duration of the spike is about 70 ms. For all plots, the spike will be located in the 0 to 1 sec plot which is the second plot in the second row from the left side of the given figure. The last plot (third row, fourth plot) located at the bottom right shows the rate of formation of phase cones averaged over eleven-second period. A red-colored contour defines the estimated area of possible epileptic activity which will be used later on to compute the spatial average of phase cones in the epileptogenic zone. The color bar indicates the rate of formation of phase cones, and it is same for all plots in this figure. These plots in Figure 3 show that the peak value exhibits an oscillatory pattern and also the spatial location of the peak slightly changes from one plot to the next. Higher phase cone activity is present in the left central and parietal areas which matches with the findings of ECoG and high-density scalp recordings given in Figure 1. In addition, the phase cone activity shown in Figure 3 is present in larger areas as compared with the spike activities shown in Figure 1. Also in the epileptogenic zone, the values are higher than the values derived from the shuffled data.

The rate of formation of phase cones in the ripple band for the spike #1 is given in Figure 4. The oscillatory pattern is visible here also. The phase cone activity is present in larger areas in each plot as compared with Figure 3 for the low gamma band. This would suggest that phase cones in the ripple band are able to pick up the interconnected neuronal activity in a larger area as compared with the same activity in the low gamma band. This is elaborated in detail in the discussion section.

#### 3.1.2. Spike #2 of the Subject #1

The phase cone activities for the spike #2 are given in Figure 5 for the low gamma band and in Figure 6 for the ripple band, respectively. For this spike, the low gamma band activity (Figure 5) is relatively much stronger and spatially distributed in a larger area as compared with the low gamma band activity of the spike #1 given in Figure 3. This possibly could be due to a larger number of neurons involved in the spike #2 as compared with the spike #1. There are multiple peaks in the top row of plots in going from −5 sec to −1 sec. After that, in the second row, there is one strong peak in the 0 to 1 sec time frame. After that, multiple peaks begin to appear in successive frames during the period of 0 to 6 sec. The peak intensity, i.e., number of phase cones, from one frame to the next, exhibit an oscillatory pattern. In addition, spatial oscillatory patterns are also noticeable in going over successive frames.

The ripple band activity for the spike #2 is given in Figure 6. Once again, similar to the spike #1, the ripple band activity is comparatively higher in each frame as compared with low gamma band activity for this spike given in Figure 5. In the left central and parietal areas, the phase cone activity is definitely related to the epileptic spike activity. However, the higher activity spots in the front central and in the left frontal areas could be due to muscle artifacts. Or, it could be related to epileptic and/or cognitive brain activity which is difficult to separate from the muscle activity. The spatial and temporal oscillatory patterns are also visible in Figure 6 which are similar to the ones shown in Figure 4 for the spike #1.

#### 3.1.3. Spike #3 for the Subject #1

The phase cone activity in the low gamma band for the spike #3 is given in Figure 7. The spatial patterns are very similar to the spike #1 and #2. There are multiple noticeable peaks before the spike during the period of −5 to 0 second. These collapse to a very strong single peak during the spike in the frame of 0 to 1 sec. Refer to the second plot from the left in the second row. After that, the phase cone activity begins to diverge in larger areas as one moves from 1 to 2 sec frame toward the 3 to 4 sec frame. Even after that, four peaks are distinguishable in the 4 to 5 sec frame. In the 5 to 6 sec frame, only two strong peaks are visible but the phase cone activity is spread in large central and parietal areas. There is some phase cone activity in the frontal area which could be related to epileptic or cognitive brain activity, and it is difficult to differentiate between one or the other without more controlled experiments.

The phase cone activity in the ripple band for the spike #3 is given in Figure 8. The activity is spread in large areas including the left central and parietal areas which are the primary areas of interest. The activity is also present in the front central area in most of the frames which may or may not be related to epileptic spike activity. In the beginning four frames between −5 and −1 sec, the phase cone activity is distributed in a large area. This begins to spatially tighten in the −1 to 0 sec frame and continues to do so in the 0 to 1 sec frame where the epileptic spike is located. The majority of the phase cone activity in the 0 to 1 sec frame is located in the left central and parietal areas which matches with the spike activity area related to EEG amplitude plot shown in Figure 1. Beyond that in Figure 8, the activity again diverges in larger areas in the frames from 1 to 6 sec. The averaged phase cone activity is given in the last frame (third row right plot) which shows three strong distinct peaks in the left central and parietal areas which are also enclosed within the red contour.

### 3.2. Subject #2

For this subject also, spatial patterns of the phase cone formations for three randomly selected spikes were analyzed and plotted. For the sake of brevity, spatial patterns for only one of the spikes are described below. However, the spatial patterns were similar for the other two spikes also. Spatial patterns of the phase cone activity for the spike #3 in the low gamma band are given in Figure 9. The subject #2 had epileptic activity in a large area spreading from the basal temporal lobe to the right temporal lobe. In spatial plots, this activity will predominantly show up in the right temporal area of the plots and this is what we are seeing (Figure 9). The predominant area is marked with a red contour in the bottom row, right plot. In all of these spatial plots, the higher phase cone activity is in the right side of the plots, i.e., right temporal area of the brain. There are other hot spots also in the left central and parietal areas. Refer to the top row, right two plots. These hot spots are probably related to other cognitive brain activities and might not be related to epileptogenic activities because in four hours of EEG data of this subject, there were no observed epileptic spike activities in the left central and parietal areas.

The ripple band activity for the spike #3 for this subject is given in Figure 10. In these plots also, the higher activity is in the right temporal central and parietal areas and also slightly more spread in larger areas as compared with plots given in Figure 9. In the top row (Figure 10), there is also recognizable phase cone activity in the left frontal and central areas which possibly could be related to other cognitive processes in the brain. There is also heightened phase cone activity in the left frontal, central, and parietal areas (second row, left and right plots, and third row 5–6 sec plot of Figure 10). These could also be due to other cognitive processes in the brain.

### 3.3. Subject #3

The spatial plots of the phase cone activity in the low gamma band for the spike #1 for the subject #3 are given in Figure 11. These plots also show higher phase cone activity in the left premotor area and in the left central and parietal midline area. The possible epileptogenic activity area is marked with a red contour (refer to the right plot in the bottom row in Figure 11). The activity is spread in larger areas in the top row of the plots during −5 to −1 sec, and it becomes focused in smaller area during 0–2 sec period and after that again in 3-4 sec time frame. Overall plots show spatial oscillatory patterns during the entire period of ±5 sec from the location of the spike.

The phase cone activity in the ripple band for the subject #3 is given in Figure 12. As compared with the low gamma band (Figure 11), the activity is spread in larger areas for the ripple band. There are very noticeable hot spots, like a pearl chain, in the right visual to lower right parietal areas. These hot spots possibly could be due to muscle artifacts originating in the right upper neck area and also on the scalp slightly below the right visual cortex.

The possible epileptic zone is marked with a red contour, and within this contour, the phase cone activity is higher in all of the spatial plots covering the ±5 sec duration from the location of the spike in the 0–1 sec time frame. The phase count variations in spatial profiles are also visible in successive frames. The quantification of these variations is described in the next section. The spatial plots for the spikes #2 and #3 were similar and for the sake of brevity not included here. However, the averaged epileptic spike activity for all three spikes for this subject is described in the next section.

### 3.4. Averaged Epileptic Spike Activity

**Figure 13:**
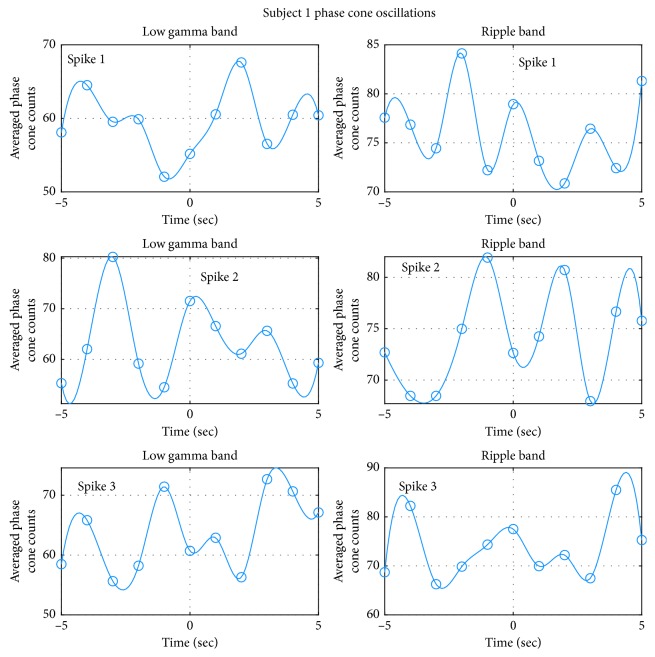
Oscillatory behavior of the averaged phase cone counts in the epileptogenic zone for the subject #1.

The number of phase cones were integrated and then divided by the area defined by the red contour. This was done for each frame in Figures 3to 12 for all three subjects. The averaged phase cone counts for the subject #1 are plotted in Figure 13. This is plotted as averaged counts versus time. The time axis goes from −5 to +5 sec with location of the epileptic spike at 0 sec. The number of averaged phase cones is slightly higher in the ripple band as compared with the low gamma band for all three spikes. A spline curve fitting was applied to data points. An oscillatory pattern with respect to time is present for all three spikes in the low gamma band and also in the ripple band.

For the low gamma band, the oscillatory pattern is visible for all three spikes. Refer to the left column of plots in Figure 13. The time period between two consecutive peaks is variable in all of these plots. Therefore, only an averaged time period was calculated for each plot. For the spike #1 (top left plot), the peaks are located at −4.4, −2, +2, and 4.5 seconds. Ignoring the broad peak in the middle, the averaged time period between the peaks is 2.45 sec. For the spike #2 (middle left plot), the peaks are located at −3, 0.3, and 3 seconds. This gives us an averaged time period of 3 sec between the peaks. For the spike #3 (bottom left plot), peaks are located at −4.5, −1, 1, and 3.2 seconds with an averaged time period of 2.63 sec between the peaks. The mean and standard deviation of averaged time periods for all three spikes in the low gamma band is: 2.69 ± 0.28 (*n* = 3) seconds.

For the spike #1 (top right plot) and #2 (middle right plot) in the ripple band, oscillations exhibit a regular pattern which is subdued for the spike #3 (bottom right plot). For the spike #1 (top right plot), clearly recognizable peaks are located at −2, 0, and 3 seconds. An averaged time period of 2.25 sec between the peaks was found for the spike #1. Similarly for the spike #2, clearly recognizable peaks are located at −1, 2, and 4.5 seconds with an averaged time period of 2.25 sec between the peaks. A similar oscillatory pattern is also present for the spike #3 but with a broad peak between −3 and +1 seconds. Recognizable peaks are located at −4.3, 0, 2, and 4.5 seconds. An averaged time period of 2.73 sec was calculated for the peaks between −5 and 5 seconds. The mean and standard deviation of averaged time periods for all three spikes in the ripple band is 2.41 ± 0.28 (*n* = 3) seconds.

**Figure 14:**
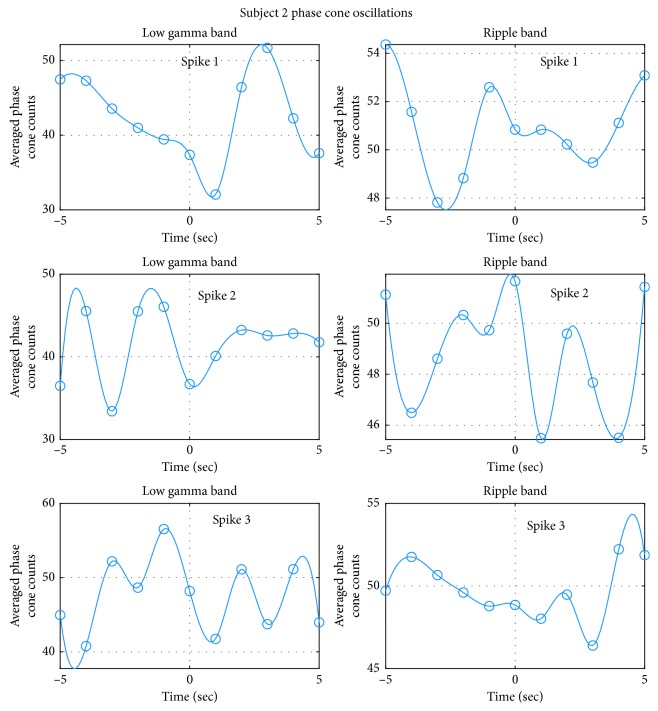
Oscillatory behavior of the averaged phase cone counts in the epileptogenic zone for the subject #2.

The oscillatory behavior of the averaged phase cone counts for the subject #2 is given in Figure 14. In the low gamma, the spike #3 has a good oscillatory behavior over the ±5 sec duration. However, in contrast, the spike #1 exhibits oscillatory behavior during the 0–5 sec period and the spike #2 exhibits oscillatory behavior during the −5 to 0 sec period. There is no complete cycle in the spike #1 (top left plot). In the spike #2 (middle left plot), there is one complete cycle with a time period of 2.7 sec. For the spike #3, there were two complete cycles with an averaged time period of 2.75 sec. The mean and standard deviation of averaged time periods for the spike #2 and #3 in the low gamma band is: 2.725 ± 0.04 (*n* = 2). Here, we have not included the spike #1 because of the lack of a complete cycle.

For the ripple band the oscillatory behavior is given in the right side plots in Figure 14. Once again, only spike #2 exhibits the oscillatory pattern. A partial oscillatory pattern is visible for the spike #1 and spike #3 without a complete cycle (top and bottom right plots in Figure 14). For the spike #3, two complete cycles can be recognized between the −2 and +3 sec period. This gives a mean and standard deviation of 2.6 ± 0.14 sec for two cycles in the spike #2 in the ripple band.

**Figure 15:**
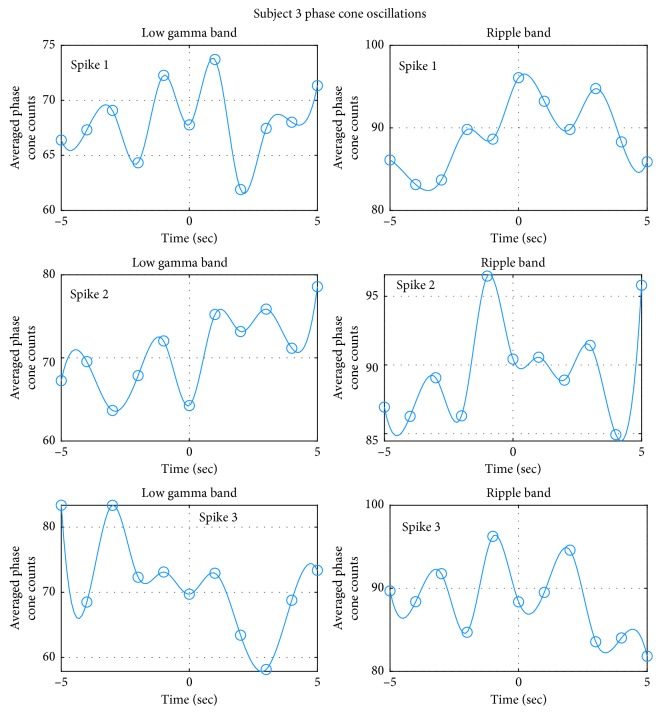
Oscillatory behavior of the averaged phase cone counts in the epileptogenic zone for the subject #3.

The oscillatory behavior of the averaged phase cone counts for the subject #3 is given in Figure 15. For this subject all three spikes show recognizable oscillatory patterns in the low gamma band and also in the ripple band. The time period between the locations of successive spikes is variable. The mean values of peak separation, i.e., time period in the low gamma band for the spike #1 is 2.37 sec, for the spike #2 is 2.53 sec, and for the spike #3 is 2.5 sec. The mean and standard deviation of averaged time periods for all three spikes in the low gamma band is 2.467 ± 0.08 (*n* = 3) seconds.

The oscillatory patterns in the ripple band (right plots in Figure 15) for the subject #3 are very similar to the patterns observed in the low gamma band (left plots, Figure 15). The mean values of peak separation, i.e., time period in the ripple band for the spike #1 is 2.5 sec, for the spike #2 is 2.1 sec, and for the spike #3 is 2.53 sec. The mean and standard deviation of averaged time periods for all three spikes in the ripple band is: 2.38 ± 0.03 (*n* = 3) seconds.

## 4. Discussion

The spatial plots given in Figures 3to 15 show that there is a higher phase cone activity in the epileptic zones as compared with nearby areas. This is observed for all three subjects. Also, the phase cone activity in the ripple band is spread in larger areas as compared with the low gamma band activity. This would imply that analysis of EEG phase cones in the ripple band picks up the activity of cortical neurons involved in the epileptic processes in a better fashion as compared with the same analysis performed in the low gamma band. This is based on the assumption that same cortical neurons are involved in the formation of the epileptic spike whether one performs the analysis in the ripple band or in the low gamma band. This conclusion is similar to what has been suggested earlier based on EEG measurements [25–27]. These authors have suggested that these high-frequency oscillations (80–250 Hz) could be seen in scalp EEG. Thus, what we are reporting here is definitely feasible.

The results related to the spatiotemporal oscillatory activity near to the epileptic spike are relatively new and are based on EEG phase cone formations. It has been suggested earlier that the EEG phase cone activity could be related to the state transitions and/or neural avalanche activity [1, 2, 13]. Thus, our results might be useful to study the oscillatory behavior of cortical neurons taking part in the formation and dissolution of an epileptic spike. We have described our results for a period ±5 sec from the location of the spike. It is a possibility that different sets of cortical neurons are taking part in the oscillatory behavior during this period of ±5 sec. For the subject #1, the averaged time period of oscillations in the low gamma band is 2.69 sec and in the ripple band is 2.41 sec. So the oscillatory activity of cortical neurons in the ripple band might be slightly faster as compared with the low gamma band oscillatory activity of cortical neurons. The same is true for the subjects #2 and #3. This might have some significance for epileptogenic cortical connectivity where speed of propagation along white matter fibers might be slightly faster in the ripple band.

The muscle artifacts are a big problem, and it is difficult to separate them from the brain (EEG) activity [28, 29]. It is particularly true for low-level cranial muscle activity where electromyographic (EMG) signal is similar or below the EEG signal level. A large amplitude muscle activity is visible in the EEG data sets and can be easily removed with the use of signal processing techniques, such as, filtering, spectral analysis, independent component analysis (ICA), or principal component analysis (PCA). Also, another confounding factor is that the frequency spectrum of EMG and EEG are very similar which makes it difficult to use signal processing techniques to separate the muscle activity from the brain activity in scalp EEG data [28, 29]. This is particularly true for the EEG signal collected on the forehead and face and at the back of the head below the visual cortex area. These electrodes do pick up the electrical activity from basal area of the cortex. In spatial plots, the contamination from these artifacts shows up at the bottom of the plot and also at the top of the plot. Development of better EMG rejection techniques will help us to analyze the electrical activity arising from the basal cortical area. The phase cone activities related to cognitive processes also show up in these spatial plots along with the epileptic activity. It would be desirable to develop technologies to separate the cognitive processes from the epileptogenic activities which will help to better localize the loci of epileptic sites in the brain from the interictal EEG data. Let us hope that these tools will become available in the near future for EEG data analysis.

## 5. Conclusions

The rate of formation of phase cones in the epileptogenic areas is higher as compared with the shuffled data. This would suggest that the observed effects are above the random noise level and possibly could be useful to localize the sites of epileptic spikes. In summary, our results show that phase cone activity derived from high-density scalp EEG data has a potential to study the oscillatory behavior of epileptic spike activity and possibly could give us an insight in the neurodynamics of cortical neurons. Our study is based on interictal data near to spikes from three subjects. Extending this to a larger patient data set should be the next logical step to duplicate and further confirm our findings.

## Data Availability

The data used to support the findings of this study are available from the corresponding author upon request.
